# Józefa Franciszka Joteyko (1866–1928)

**DOI:** 10.1007/s00415-019-09512-9

**Published:** 2019-08-20

**Authors:** Katarzyna Pekacka-Falkowska, Anna Maria Pekacka-Egli

**Affiliations:** 1 Poznan University of Medical Sciences, Przybyszewskiego 37a, 60365 Poznan, Poland; 2 Zürcher Reha Zentrum Wald, Faltigbergstrasse 7, 8636 Wald, Switzerland

## Józefa Franciszka Joteyko (1866–1928)

**Fig. 1:**
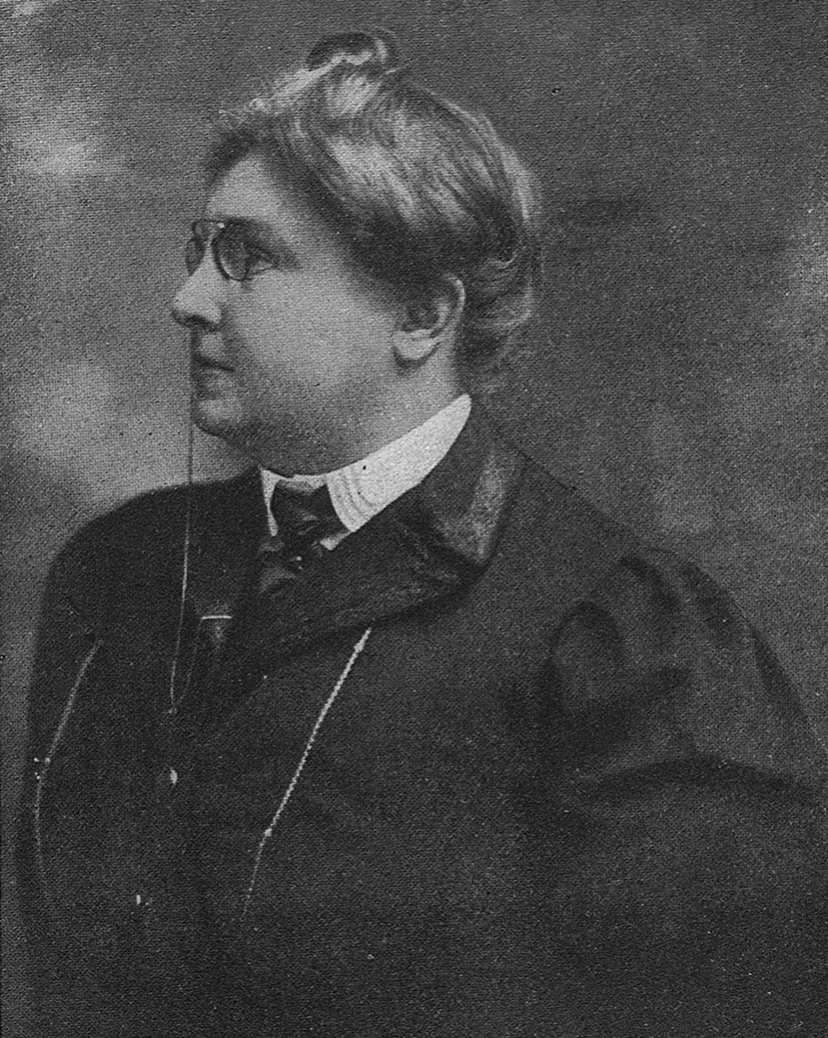
Józefa F. Joteyko (by courtesy of the Main Library of Poznan University of Medical Sciences)

In the early twentieth century, Józefa Franciszka Joteyko (known to a wider public as Joséphine Joteyko) (Fig. 1) was the most famous Polish woman scientist in Europe, next to Marie Skłodowska-Curie. She was born on 29 January 1866, on the Poczujki landed estate near Kiev, Ukraine, into a family of Polish landowners. In 1873, her family moved to Congress Poland (then under Russian rule) to seek an education for their four children.

After completing her secondary education at a boarding school in Warsaw and through private tuition, the 20-year-old Joteyko enrolled, in 1886, at the University of Geneva to study the Natural Sciences and was awarded a bachelor degree in 1888. Next, she moved to the Free University of Brussels to study Medicine, which she was to continue in Paris at the Sorbonne. Her doctoral thesis (1896) on muscle fatigue and respiration [1], written under the direction of Charles Richet, a future Nobel Prize Winner, was awarded a special prize by the faculty authorities.

After two busy years in France, and tired of her responsibilities as a GP, Joteyko returned to Belgium where she started scientific work as an assistant at the Solvay Physiological Institute. One of her collaborators was a Polish neuropathologist, Michalina Stefanowska, who was later to become her life partner. The two women published together numerous research papers on neurology and the neurobiology of pain and fatigue [2, 3], earning several prizes, for example the Dieudonnée Prize from the Belgian Royal Academy of Medicine (1901) and the Montyon Prize from the French Academy of Sciences (1903). Moreover, Joteyko, in 1903, in recognition of her excellence in research into the physiology of the nervous system was appointed director of the Solvay Laboratory. Meanwhile, she also lectured on Experimental Psychology at the Free University of Brussels and on Pedagogical Psychology at teacher seminars in Charleroi and Mons.

In 1905, the 39-year-old Pole became President of the Belgian Neurological Society and was elected chairman at the First Belgian Congress of Neurology and Psychiatry [4]. Although Joteyko was not a practicing neurologist, in her research she had focused on the psychophysiology of the nervous system and her works had generated significant interest, in particular amongst medical scholars in Europe and further afield. In Belgium, she also started to develop her interest in experimental paedology, the study of children’s behaviour and development, that fused, among other things, neurology and pedagogical issues. In 1908, in Geneva, she initiated the quarterly “Revue Psychologique” and served as its editor-in chief for the next 6 years. She also organized the first International Congress of Paedology in Brussels, in 1911, and the following year founded the International Faculty of Paedology at the Free University of Brussels.

During the First World War, following the German occupation of Belgium Joteyko moved to Paris, where her former teacher Richet put his laboratory at her disposal. In 1916, she was appointed, as the first woman in history, professor at the College de France. In the years 1916–1918, in acknowledgment of her merits and innovative research, she was awarded numerous prizes from prestigious organizations like the French Academy of Sciences, Académie Nationale de Médecine and the Collège de France.

With the proclamation of Poland’s independence, in 1918, Joteyko decided to return to her homeland. There, unable to obtain a university chair because of gender-based discrimination, she engaged in the activities of the newly established National Institute of Pedagogy and started lectures in Experimental Psychology at the University of Warsaw. As a result of the restructuring of the Polish educational system, in 1925, the Pedagogical Institute was closed and Joteyko lost her temporary job. However, thanks to her colleagues, shortly thereafter she became an honorary docent at Warsaw University’s medical department and at the Institute for Social Pedagogy. At that time, she also engaged herself in establishing special needs education institutions in Poland. Moreover, she joined the editorial boards of the journals the “Polish Archive of Psychology” and the “Polish Pedagogical Yearbook”.

Her short life in the newly independent Poland was, however, far from ideal. Joteyko’s work in Warsaw neither gave her an intellectual satisfaction, nor enabled her to develop fully her research activities and international collaboration, and this as a result of financial limitations and a lack of institutional support. In the mid-1920s she developed a cardiac disease. Her health deteriorated in 1927, and shortly thereafter, on April 24th, 1928, she passed away.

Joteyko’s scientific activity had three foci: neurophysiology, psychology and pedagogy. All her works concerning neurophysiology were devoted to the issue of fatigue and exhaustion of the neuromuscular system. Among others, she explained a number of clinical symptoms in the muscular system, for instance the reaction of degeneration of muscle contractures, by the properties of sarcoplasm [5], the asymmetry in the distribution of cutaneous sensors for pain [6], and the dissociation between tactile sensation and pain during anesthesia [3]. Together with Charles Henry, she also studied maximal voluntary activation and central fatigue, and developed a mathematical formula for the ergographic fatigue curve [7]. Apart from the careful analysis of the biological mechanisms responsible for experiencing fatigue, Joteyko also tried to unravel the psychological aspects of pain sensations. Consequently, she considered the question of physical, psychic and moral suffering which was inflicted by pain, and investigated the relationship between the threshold of pain and fatigue itself [8]. Finally, she integrated neurology into her paedological research, attempting to link the anatomy and the physiology of the nervous system with the numerous aspects of a child’s development [9], therefore pioneering in developmental neurology and developmental cognitive neurosciences.

Joteyko’s first and beloved teacher, Richet, said once that he had never had a student more intelligent and more industrious than ‘the Pole’ [10]. Yet, Joteyko was not only a brilliant woman scientist, she was also an emancipated, uncompromising woman who gave up her career in western Europe for patriotic idealism and, as it turned out, dashed hopes.
